# Design and Synthesis of Cobalt-Based Hollow Nanoparticles through the Liquid Metal Template

**DOI:** 10.3390/mi13081292

**Published:** 2022-08-11

**Authors:** Yuan Ji, Zhenlong Li, Yundan Liu, Xianghua Wu, Long Ren

**Affiliations:** 1 State Key Laboratory of Advanced Technology for Materials Synthesis and Processing, International School of Materials Science and Engineering, Wuhan University of Technology, Wuhan 430070, China; 2 School of Basic Medical Sciences, Zhuhai Campus, Zunyi Medical University, Zhuhai 519041, China; 3 Hunan Key Laboratory of Micro-Nano Energy Materials and Devices, Laboratory for Quantum Engineering and Micro-Nano Energy Technology, Faculty of Materials and Optoelectronic Physics, Xiangtan University, Xiangtan 411105, China

**Keywords:** liquid metal, hollow structure, transition-metal compounds, electrocatalyst

## Abstract

Co-based compounds have attracted much attention due to their competitive catalytic activities. To enhance their intrinsic electrocatalytic activity, morphology engineering is one of the effective strategies. Hollow structures have fascinating properties due to their low density and high loading capacity. In this work, we introduce a Ga-based liquid alloy as a reactive template for the synthesis of varying Co-based hollow nanoparticles. The fluidity character of the Ga-based liquid alloy facilitates the large-scale production of nanoparticles via a top-down shearing process. The pre-installed active species (here is Zn) in the liquid alloy serve as a sacrificial source to quantitatively reduce Co^2+^ ions and form Co-based compounds. Well-structured Ga/CoOOH core-shell nanospheres are thus successfully prepared, and more varied Co-based hollow nanoparticles can be obtained by post-treatment and reaction. Hollow structures can offer enhanced interfacial area and increased active sites, benefiting the catalytic performance. Among the prepared Co-based catalysts, CoSe_2_ hollow nanoparticles exhibited the best oxygen evolution reaction (OER) activity with an overpotential of 340 mV at the current density of 10 mA/cm^2^. This work provides a novel strategy for the rational design and simple preparation of hollow nanoparticles.

## 1. Introduction

As a class of earth-abundant materials, Co-based compounds are widely used as electrocatalysts owning to their innate electrochemical activity and tunable properties [1,2,3,4]. To further improve the catalytic activities, many efforts have been devoted to designing unique morphologies to increase active sites and enhance charge transport capability. Among these morphologies, the hollow structures possess a large surface area, low density, and high loading capacity to promote the accessibility of active sites [5,6,7]. For example, Fan et al. prepared metal-organic framework derived hollow CoSe_2_ microspheres and found the well-distributed hollow structures obtained via adjusting the annealing temperature exhibited outstanding electrochemical performance [8]. The template method, including soft, hard, and self-sacrifice templates, is an effective strategy to construct a hollow structure [5,9]. Conventionally, the templates for synthesizing hollow structures are commonly solid, such as SiO_2_, PVP (polyvinylpyrrolidone), carbonaceous, and so on [8,10,11,12]. Inevitably, these approaches would involve multistep processes, including the preparation of the template and the following deposition of target materials; this increases the complexity of design and the risk of lacking uniformity and universality.

Room temperature Ga-based liquid metals (LMs) have attracted attention in many fields due to the ideal combination of metallic properties and fluidic properties [13,14,15]. Benefited from the richly surface chemical activity and good fluidity, Ga-based LMs can serve as novel soft templates for the preparation of low-dimensional materials. Through applying chemical reactions at the LM-solution interface, the preparation of various nanomaterials can also be achieved by changing the reactive surroundings [16,17,18,19,20]. For example, Ghasemian et al. prepared atomically thin hydrated MnO_2_ using a galvanic replacement reaction between permanganate ions and Ga [21]. Meanwhile, based on the fluidity of LMs, the metal droplets can be easily sheared into nanoparticles and these nanoparticles simultaneously complete the construction of large-scale shell-core structures through interfacial deposition [22]. Nevertheless, the diversity of products is still limited if the related reaction is only between Ga and other species. For instance, it is kinetically slow for the reaction between Co^2+^ ions and Ga^0^ (which is actually thermodynamic favored), due to their redox potential difference being quite close [23]. It was found that eutectic metal elements inside the Ga-based LMs would undergo thermodynamically selective self-limiting surface oxidation at the LM-air interface, offering the successful synthesis of varying 2D oxides [24]. Similarly, it is believed that the higher reactive activity of Ga-based LMs can be tuned by adding more reactive metals inside the liquid matrix; more reactions with a greater variety of products can be achieved, such as the reduction of Co^2+^ ions.

In this work, we successfully synthesized varying Co-based hollow nanostructures by setting Ga-Zn liquid alloys as the soft templates. The experimental results showed that Ga-Zn alloys are more reactive and their uniformly distributed nanoparticles are easily obtained by ultrasound. It indicates that uniform templates can be collected and large-scale production can be achieved. As expected, through subsequent treatment of the precursor, the hollow nanoparticles are evenly distributed and possess good electrocatalytic performances. The obtained hollow CoSe_2_ catalyst exhibited good OER activity in an alkaline solution.

## 2. Materials and Methods

### 2.1. Chemicals

The Ga 99.99%, Zn 99.99%, Co(NO_3_)_2_·6H_2_O 99.99%, selenium powder 99.9%, 200 meshes, ammonia solution (NH_3_ 25~28%), and thiourea 99% are purchased from Aladdin Chemistry Co., Ltd. (China).

### 2.2. Preparation of Hollow CoSe_2_ Nanospheres

Preparation of CoOOH/Ga: a 700 mg GaZn liquid alloy was added into a 30 mL ethylene glycol solution and ultrasonicated with a cell crusher for 20 min to obtain GaZn nanoparticles. The nanoparticles were collected by centrifugation at 1000 to 3000 rpm for particle size screening. The GaZn nanoparticles were added dropwise into the 10 mM Co solution and stand for 10 min. The samples were collected by washing with deionized water and centrifugation, then freeze-dried in a freeze dryer.

Preparation of Co(OH)_2_: 200 μL ammonia water was diluted with 20 mL deionized (pH~11.3) water and stirred evenly; then, 20 mg CoOOH/Ga was put into the solution and left to stand for 36 h to obtain a powdery white precipitate. The samples were collected by centrifugation with deionized water and then freeze-dried in a freeze dryer.

Preparation of CoSe_2_: the obtained 10 mg Co(OH)_2_ powder was placed in the center of the tube furnace, and 50 mg of selenium powder was placed upstream of the tube furnace. Selenization annealing was carried out at 350 °C and 450 °C for 1 h to obtain the black powder, and the samples were collected.

Preparation of CoS_2_: the obtained 10 mg Co(OH)_2_ powder was placed in the center of the tube furnace, and 50 mg thiourea was placed upstream of the tube furnace. The sulfidation annealing temperature was maintained at 350 °C for 1 h.

### 2.3. Electrochemical Measurements

Electrochemical measurements are performed by an electrochemical workstation (CHI 660D, CH Instruments, Inc., China) in a 1M KOH (Aladdin Chemistry Co., Ltd., China) solution (pH~13.8); a typical three-electrode electrochemical cell consists of a reference electrode (using Hg/HgO electrode), a counter electrode (carbon rod), and a working electrode. Convert the potential to a reversible hydrogen electrode (RHE) using a standard conversion formula with 95% iR compensation. The electrocatalytic OER performance was measured by linear sweep voltammetry (LSV) curves at a scan rate of 5 mV/s under ambient nanospheres. Electrochemical impedance spectroscopy was performed at an open-circuit voltage in the frequency range 10^0^–10^6^ Hz. The electrochemical surface area was obtained by scanning CV with a voltage window of 1.532–1.567 V vs. RHE in a KOH solution at scan rates from 20 to 180 mV/s.

## 3. Results and Discussion

**Figure 1:**
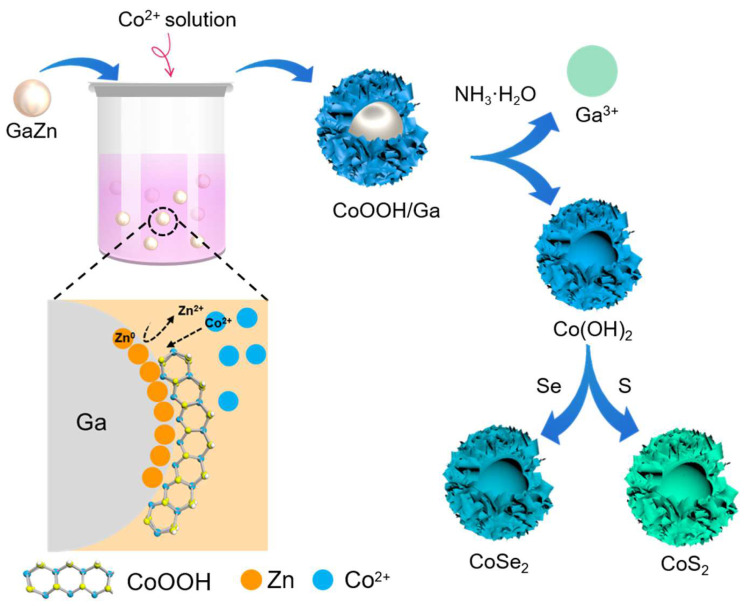
Schematic illustration of the formation for the Co-based compounds.

Figure 1 shows a schematic illustration for the synthesis of Co-based hollow nanoparticles. The prepared GaZn LM nanoparticles gained by ultrasonication are dropped into the solution containing Co^2+^, and then the galvanic reaction happens at the interface of LM and aqueous solution. The redox reaction actually involved two species: one is the Co^2+^ in the solution that can move to the LM interface; and the other is the internal Zn atoms, which can also move freely to the surface of LM due to the dynamical atomic arrangement inside LM. The main reason is that the Zn has a lower electrochemical potential (E^0^_Zn/Zn_^2+^ = −0.763) than Ga (E^0^_Ga/Ga_^3+^ = −0.549) and is more likely to react with Co ions (E^0^_Co/Co_^2+^ = −0.280) [23]. The more reactive atoms tend to move to the surface and participate in redox reactions. Therefore, the Ga nanoparticles are retained in the core, while the Zn atoms in LM alloys undergo galvanic replacement reactions with Co ions to form solid products wrapped around the surface of Ga nanoparticles. Further, since Ga atoms can react with an alkaline solution to convert to Ga^3+^, the inner Ga core can be removed to obtain Co-based hollow nanoparticles. By means of a following post-processing reaction, more diversity can be achieved; for instance, CoS_2_ and CoSe_2_ hollow nanoparticles can be obtained by the following sulfurization and selenization treatment.

**Figure 2:**
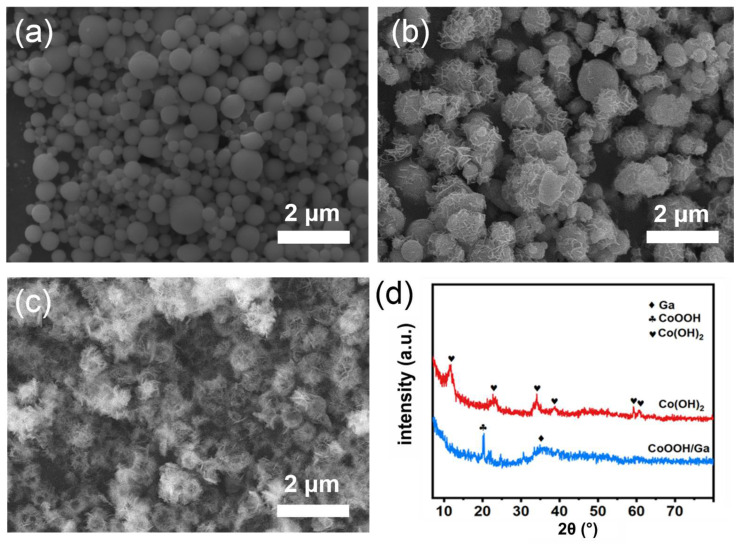
SEM images of (**a**) GaZn nanoparticles; (**b**) CoOOH/Ga; (**c**) Co(OH)_2_; and (**d**) the XRD patterns of CoOOH/Ga and Co(OH)_2_.

The morphologies of the samples after a galvanic reaction were characterized by SEM images, as shown in Figure 2a and Figure S1b. It was observed that the GaZn alloy nanoparticles were obtained by ultrasound in ethylene glycol solution. The nanoparticles are uniformly dispersed and have a smooth surface. The surrounding ethylene glycol can prevent the formation of a surface Ga-oxides shell. Figure S1a plots the particle size distributions of nanoparticles obtained in Figure 1 between 200 and 400 nm. After reacting with the Co ion solution, the nanosheets are found to wrap on the spherical surface of nanoparticles, forming core-shell nanostructures (Figure 2b and Figure S1c). For comparison, pure Ga nanoparticles without Zn contents were also injected into a Co ion solution. The obtained products exhibit a smooth surface, as demonstrated in the SEM image in Figure S2; this indicates that no obvious reaction happened between the Ga and Co ions. This clear difference claims that the introduction of the Zn element in the LM matrix triggered the reaction and the formation of a uniform shell with sheet-like nanostructures. This also means quantitative deposition of the Co-based compound as the shell can be achieved by setting a specific amount of Zn inside the Ga LM matrix. The crystal structure of the material was further analyzed by XRD. Figure 2d shows that the core-shell structures obtained by the replacement reaction of a GaZn alloy with Co ions correspond to the peaks of CoOOH [25]. Considering the Ga LM nanoparticles are amorphous, the CoOOH should correspond to the outer shell. Here, due to the relatively high reactivity of Zn element, the Co ions can be reduced by Zn atoms in solution [26]. In the presence of oxygen and OH^−^ in solution, the Co^2+^ can release electrons under the applied bias to form CoOOH [27].

The Ga/CoOOH core-shell nanoparticles remain stable in a neutral solution, while after the alkaline solution treatment, Ga would be oxidized to cationic into the solution and removed from the inner core. Thus, Co-based hollow nanoparticles can be obtained after Ga is completely removed, as evidenced in Figure 2c and Figure S1d. The XRD pattern in Figure 2d illustrated that the hollow nanoparticles in Figure 2c correspond to Co(OH)_2_. It indicates that the outer CoOOH shell was converted to Co(OH)_2_ through dissolution-crystallization processes in ammonia solution [28]. It should be noted that different mass ratios of CoOOH/Ga will affect the sample morphology and the consumption of the Ga core in an ammonia solution. Figure S3a,b show the SEM images of Co(OH)_2_/Ga-40 (Figure S3a) and Co(OH)_2_/Ga-60 (Figure S3b) obtained by adding 40 mg and 60 mg of CoOOH/Ga into a 20 mL ammonia solution with pH~11.3, respectively. It was observed that the hollow structure cannot be maintained and large-sized residual Ga nanoparticles remained. It seems Ga cannot be completely consumed due to the increase in the mass of the precursor. The different pH of the alkaline solution also affects the morphology of the sample after removing the LM templates. When the pH is~12.3, the morphology of the samples is partly kept hollow and partly transformed into flakes (Figure S4a,b). Moreover, the strong alkaline solution would result in the construction of hollow structures during the removal process of the Ga template. The samples obtained by soaking the CoOOH/Ga precursor in a NaOH solution with pH~11.3 are sheet-like structures (Figure S4c,d). It is believed that the rapid depletion of Ga atoms in the strong alkaline solution could increase the surface tension and induce the rupture of the outer shell.

**Figure 3:**
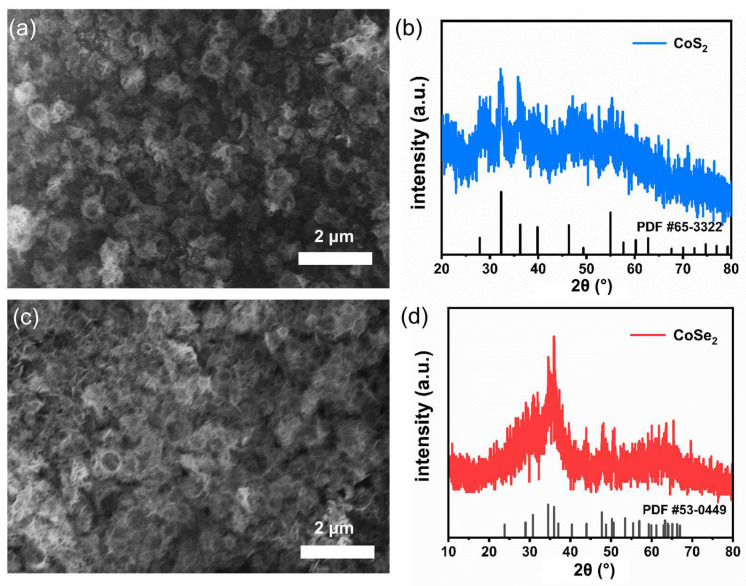
The SEM images of (**a**) CoS_2_ and (**c**) CoSe_2_; the XRD patterns of (**b**) CoS_2_ and (**d**) CoSe_2_.

The obtained hollow Co(OH)_2_ nanoparticles can be used as good templates for the preparation of other Co-based materials. Here, serving as representative examples, hollow CoS_2_ and CoSe_2_ nanostructures were successfully synthesized via further sulfurization and selenization treatments, respectively. As shown in Figure 3a and Figure S5a, the morphology of CoS_2_ after 350 °C sulfurization still maintains a good hollow nanostructure, being composed of uniformed thin nanosheets. The XRD (X-ray Diffraction) analysis in Figure 3b claimed that the as-prepared product corresponds to CoS_2_ (standard card PDF #65-3322). Hollow CoSe_2_ nanoparticles (Figure 3c and Figure S5b) can be also obtained by selenizing the hollow Co(OH)_2_ nanoparticles at 350 °C. The XRD result of the sample also confirms its crystalline structure is CoSe_2_ (standard card PDF #53-0449, Figure 3d). Note, it is better to use mild condition for further treatment. For example, when the selenization temperature was raised to 450 °C, the hollow structures collapsed into fragmented nanosheets (Figure S6).

**Figure 4:**
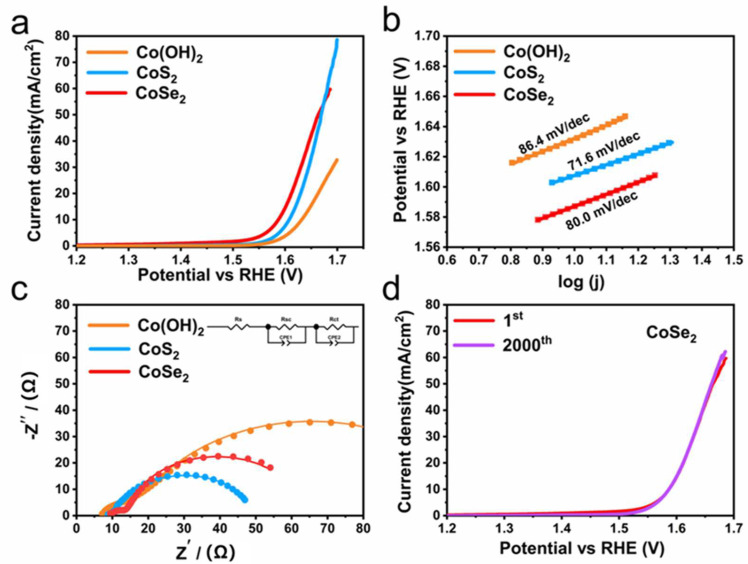
(**a**) OER polarization curves of Co(OH)_2_, CoS_2_, and CoSe_2_; (**b**) Tafel plots; (**c**) Nyquist plots; (**d**) polarization curves of CoSe_2_ before and after 2000 cycles.

The OER activity of the as-prepared samples was tested through a typical three-electrode system in 1M KOH, and the performances of different Co-based hollow nanoparticles were evaluated. The LSV curves show that the hollow-structured CoSe_2_ nanoparticles exhibit the best OER performance. Its overpotential corresponds to a voltage of 340 mV at a current density of 10 mA/cm^2^, which outperforms those of CoS_2_ (370 mV) and Co(OH)_2_ (402 mV). Figure 4b reveals the Tafel slopes of CoSe_2_ (80.0 mV/dec), CoS_2_ (71.6 mV/dec), and Co(OH)_2_ (86.4 mV/dec), respectively. CoS_2_ exhibited the smallest Tafel slope, which indicates that it had the fastest reaction kinetics. To characterize the electrode kinetics, electrochemical impedance spectroscopy was tested in Figure 4c. To characterize the electrode kinetics, electrochemical impedance spectroscopy was tested in Figure 4c. The charge transfer resistance (R_ct_) is obtained from the high frequency region. The semi-circular diameter of CoSe_2_ and CoS_2_ is much smaller than that of Co(OH)_2_; thus, they have a smaller R_ct_ and faster electron transport. Figure 4d tests the stability of the samples by comparing the polarization curves before and after CV (Cyclic Voltammetry, CHI 660D, CH Instruments, Inc., China) 2000 cycles of CoSe_2_. It is found that the two polarization curves almost overlap, indicating the good stability of the as-prepared CoSe_2_ as electrocatalysts.

The electrochemical surface area (ECSA) was measured by electrochemical double-layer capacitance (C_dl_). Figure S7a–c tested the cyclic voltammetry curves of Co(OH)_2_, CoS_2_, and CoSe_2_ at different scan rates. In Figure S7d, the C_dl_ values of different samples were calculated according to the CV curves, in which CoS_2_ (9.1 mF/cm^2^) has more electrochemically active sites than CoSe_2_ (6.0 mF/cm^2^) and Co(OH)_2_ (5.6 mF/cm^2^). Figure S8 shows the OER performance of CoSe_2_ at different annealing temperatures. Compared with the hollow structure, the sheet-like structure exhibits higher overpotential (400 mV) in Figure S8a and a larger Tafel slope (93.7 mV/dec) in Figure S8b. In addition, Figure S9 shows that the morphology and residual Ga particles affect the OER performance of the samples. The performances of CoOOH/Ga nanoparticles of different quality placed in alkaline solution were compared. Figure S9a measured the LSV curves of the added 20, 40, and 60 mg CoOOH/Ga nanoparticles. The overpotentials of the three samples were 402 mV (Co(OH)_2_), 430 mV (Co(OH)_2_/Ga-40), and 470 mV (Co(OH)_2_/Ga-60) at 10 mA/cm^2^, respectively. As the Ga nanoparticles content increases, the Tafel slope of the samples also increases as shown in Figure S9b. The results show that the more remaining Ga particles will reduce the OER performance.

## 4. Conclusions

In conclusion, the Zn species in the GaZn alloy nanoparticles have a lower redox potential; thus, it is easy to undergo a redox reaction for the reduction of Co ions. Taking advantage of this strategy, the Ga/CoOOH core-shell nanostructure was successfully prepared; in addition, the Co(OH)_2_ hollow nanostructure can be obtained by gently and effectively removing the Ga nanoparticles template. Furthermore, a series of other Co-based compounds can be obtained by subsequent processing. Such hollow nanostructures are able to enhance the catalytic active site and mass transport for Co-based electrocatalysts. Among the as-prepared samples, the CoSe_2_ hollow nanostructure exhibits a high catalytic activity of 340 mV at 10 mA/cm^2^ with a Tafel slope of 80.0 mV/dec. This work extends the utilization of Ga-based LMs as soft templates. This methodology can achieve the deposition of target products only relying on the electrochemical driving force. It offers the possibility to explore other hollow compounds in future research and is expected to be applied in sensing, electronics, and other fields. The unique properties of liquid metals provide a facile method for the preparation of hollow materials.

## Data Availability

Not applicable.

## Supplementary Materials

The following supporting information can be downloaded at: https://www.mdpi.com/article/10.3390/mi13081292/s1, Figure S1: (a) The particle size distributions obtained from Figure 1; the larger magnification SEM images of (b) GaZn nanoparticles; (c) CoOOH/Ga; (d) Co(OH)_2_; Figure S2: The SEM image of pure Ga nanoparticles reacting with Co solution; Figure S3: The SEM images of different quality CoOOH/Ga nanoparticles placed in ammonia solution with pH~11.3: (a) Co(OH)_2_/Ga-40; (b) Co(OH)_2_/Ga-60; Figure S4: The SEM images of (a,b) the sample of CoOOH/Ga template was cleaned by ammonia solution with pH~12.3; (c,d) the samples of CoOOH/Ga were cleaned by NaOH solution with pH~11.3; Figure S5: the larger magnification SEM images of (a) CoSe_2_ and (b) CoS_2_; Figure S6: SEM image of CoSe_2_-450; Figure S7: Electrochemical capacitance measurements with different scan rates from 60 to 180 mV/s in a 1 M KOH solution of: (a) Co(OH)_2_; (b) CoS_2_; (c) CoSe_2_; (d) the double layer capacitance (C_dl_) results for all the samples; Figure S8: (a) The OER polarization curves; and (b) Tafel plots of CoSe_2_ and CoSe_2_-450; Figure S9: (a) The OER polarization curves; and (b) Tafel plots of Co(OH)_2_, Co(OH)_2_/Ga-40, and Co(OH)_2_/Ga-60.

## References

[1] Li H., Qian X., Zhu C., Jiang X., Shao L., Hou L. (2017). Template synthesis of CoSe_2_/Co_3_Se_4_ nanotubes: Tuning of their crystal structures for photovoltaics and hydrogen evolution in alkaline medium. J. Mater. Chem. A.

[2] Feng S., Yang L., Zhang Z., Li Q., Xu D. (2019). Au-decorated CoOOH nanoplate hierarchical hollow structure for plasmon-enhanced electrocatalytic water oxidation. ACS Appl. Energy Mater..

[3] Huang Y., Zhao X., Tang F., Zheng X., Cheng W., Che W., Hu F., Jiang Y., Liu Q., Wei S. (2018). Strongly electrophilic heteroatoms confined in atomic CoOOH nanosheets realizing efficient electrocatalytic water oxidation. J. Mater. Chem. A.

[4] Zhou K.L., Wang C., Wang Z., Han C.B., Zhang Q., Ke X., Liu J., Wang H. (2020). Seamlessly conductive Co(OH)_2_ tailored atomically dispersed Pt electrocatalyst with a hierarchical nanostructure for an efficient hydrogen evolution reaction. Energy Environ. Sci..

[5] Zhou L., Zhuang Z., Zhao H., Lin M., Zhao D., Mai L. (2017). Intricate Hollow Structures: Controlled Synthesis and Applications in Energy Storage and Conversion. Adv. Mater..

[6] Kim E.H., Reddy D.A., Lee H., Jeong S., Kumar D.P., Song J.K., Lim M., Kim T.K. (2019). Hollow CoSe_2_ nanocages derived from metal–organic frameworks as efficient non-precious metal co-catalysts for photocatalytic hydrogen production. Catal. Sci. Technol..

[7] Dai C., Tian X., Nie Y., Tian C., Yang C., Zhou Z., Li Y., Gao X. (2017). Successful synthesis of 3D CoSe_2_ hollow microspheres with high surface roughness and its excellent performance in catalytic hydrogen evolution reaction. Chem. Eng. J..

[8] Liu X., Liu Y., Fan L.-Z. (2017). MOF-derived CoSe_2_ microspheres with hollow interiors as high-performance electrocatalysts for the enhanced oxygen evolution reaction. J. Mater. Chem. A.

[9] Liang L., Li J., Zhu M., Li Y., Chou S., Li W. (2019). Cobalt chalcogenides/cobalt phosphides/cobaltates with hierarchical nanostructures for anode materials of lithium-ion batteries: Improving the lithiation environment. Small.

[10] Yang S.H., Park G.D., Kim J.K., Kang Y.C. (2021). New strategy to synthesize optimal cobalt diselenide@hollow mesoporous carbon nanospheres for highly efficient hydrogen evolution reaction. Chem. Eng. J..

[11] Xiong S., Zeng H.C. (2012). Serial ionic exchange for the synthesis of multishelled copper sulfide hollow spheres. Angew. Chem. Int. Ed. Engl..

[12] Zhang H., Yu M., Song H., Noonan O., Zhang J., Yang Y., Zhou L., Yu C. (2015). Self-Organized Mesostructured Hollow Carbon Nanoparticles via a Surfactant-Free Sequential Heterogeneous Nucleation Pathway. Chem. Mater..

[13] Kim D., Thissen P., Viner G., Lee D.W., Choi W., Chabal Y.J., Lee J.B. (2013). Recovery of nonwetting characteristics by surface modification of gallium-based liquid metal droplets using hydrochloric acid vapor. ACS Appl. Mater. Interfaces.

[14] Tang J., Kumar P.V., Scott J.A., Tang J., Ghasemian M.B., Mousavi M., Han J., Esrafilzadeh D., Khoshmanesh K., Daeneke T. (2022). Low Temperature Nano Mechano-electrocatalytic CH4 Conversion. ACS Nano.

[15] Goff A., Aukarasereenont P., Nguyen C.K., Grant R., Syed N., Zavabeti A., Elbourne A., Daeneke T. (2021). An exploration into two-dimensional metal oxides, and other 2D materials, synthesised via liquid metal printing and transfer techniques. Dalton Trans..

[16] Wang S., Mao Q., Ren H., Wang W., Wang Z., Xu Y., Li X., Wang L., Wang H. (2022). Liquid Metal Interfacial Growth and Exfoliation to Form Mesoporous Metallic Nanosheets for Alkaline Methanol Electroreforming. ACS Nano.

[17] Wang Y., Mayyas M., Yang J., Tang J., Ghasemian M.B., Han J., Elbourne A., Daeneke T., Kaner R.B., Kalantar-Zadeh K. (2020). Self-Deposition of 2D Molybdenum Sulfides on Liquid Metals. Adv. Funct. Mater..

[18] Zavabeti A., Zhang B.Y., de Castro I.A., Ou J.Z., Carey B.J., Mohiuddin M., Datta R., Xu C., Mouritz A.P., McConville C.F. (2018). Green Synthesis of Low-Dimensional Aluminum Oxide Hydroxide and Oxide Using Liquid Metal Reaction Media: Ultrahigh Flux Membranes. Adv. Funct. Mater..

[19] Wang Y., Wang S., Chang H., Rao W. (2020). Galvanic Replacement of Liquid Metal/Reduced Graphene Oxide Frameworks. Adv. Mater. Interfaces.

[20] Aukarasereenont P., Goff A., Nguyen C.K., McConville C.F., Elbourne A., Zavabeti A., Daeneke T. (2022). Liquid metals: An ideal platform for the synthesis of two-dimensional materials. Chem. Soc. Rev..

[21] Ghasemian M.B., Mayyas M., Idrus-Saidi S.A., Jamal M.A., Yang J., Mofarah S.S., Adabifiroozjaei E., Tang J., Syed N., O’Mullane A.P. (2019). Self-Limiting Galvanic Growth of MnO_2_ Monolayers on a Liquid Metal—Applied to Photocatalysis. Adv. Funct. Mater..

[22] Ren L., Cheng N., Man X., Qi D., Liu Y., Xu G., Cui D., Liu N., Zhong J., Peleckis G. (2021). General Programmable Growth of Hybrid Core-Shell Nanostructures with Liquid Metal Nanodroplets. Adv. Mater..

[23] Falchevskaya A.S., Prilepskii A.Y., Tsvetikova S.A., Koshel E.I., Vinogradov V.V. (2021). Facile Synthesis of a Library of Hollow Metallic Particles through the Galvanic Replacement of Liquid Gallium. Chem. Mater..

[24] Zavabeti A., Ou J.Z., Carey B.J., Syed N., Orrell-Trigg R., Mayes E.L.H., Xu C., Kavehei O., O’Mullane A.P., Kaner R.B. (2017). A liquid metal reaction environment for the room-temperature synthesis of atomically thin metal oxides. Science.

[25] Chen Z., Kronawitter C.X., Yeh Y.-W., Yang X., Zhao P., Yao N., Koel B.E. (2017). Activity of pure and transition metal-modified CoOOH for the oxygen evolution reaction in an alkaline medium. J. Mater. Chem. A.

[26] Chen H.-Y., Chang Y.-C., Lee J.-F., Pao C.-W., Huang H.-C., Wang C.-H. (2021). Operando Identification of Hydrangea-like and Amorphous Cobalt Oxyhydroxide Supported by Thin-Layer Copper for Oxygen Evolution Reaction. ACS Sustain. Chem. Eng..

[27] Wang H.Y., Hung S.F., Chen H.Y., Chan T.S., Chen H.M., Liu B. (2016). In Operando Identification of Geometrical-Site-Dependent Water Oxidation Activity of Spinel Co3O4. J. Am. Chem. Soc..

[28] Huang J., Chen J., Yao T., He J., Jiang S., Sun Z., Liu Q., Cheng W., Hu F., Jiang Y. (2015). CoOOH Nanosheets with High Mass Activity for Water Oxidation. Angew. Chem. Int. Ed. Engl..

