# Supplementary material for: Design and Synthesis of Cobalt-Based Hollow Nanoparticles through the Liquid Metal Template
Source: Micromachines (Basel). 2022 Aug 11;13(8):1292. doi: 10.3390/mi13081292 (PMC9415925; doi:10.3390/mi13081292)
Supplement: Supplementary file 1 [file micromachines-13-01292-s001.zip › micromachines-1835426-supplementary.pdf]

Supplementary Material

Design and Synthesis of Cobalt-Based Hollow Nanoparticles through the Liquid Metal Template

Yuan Ji [1], Zhenlong Li [2,*], Yundan Liu [3], Xianghua Wu [1] and Long Ren [1,*]

[1] State Key Laboratory of Advanced Technology for Materials Synthesis and Processing, International School of Materials Science and Engineering, Wuhan University of Technology, Wuhan 430070, China
[2] School of Basic Medical Sciences, Zhuhai Campus of Zunyi Medical University, Zhuhai 519041, China
[3] Hunan Key Laboratory of Micro-Nano Energy Materials and Devices, Laboratory for Quantum Engineering and Micro-Nano Energy Technology, and Faculty of Materials and Optoelectronic Physics, Xiangtan University, Xiangtan 411105, China
* Correspondence: zlli@zmu.edu.cn (Z.L.); renlong@whut.edu.cn (L.R.)

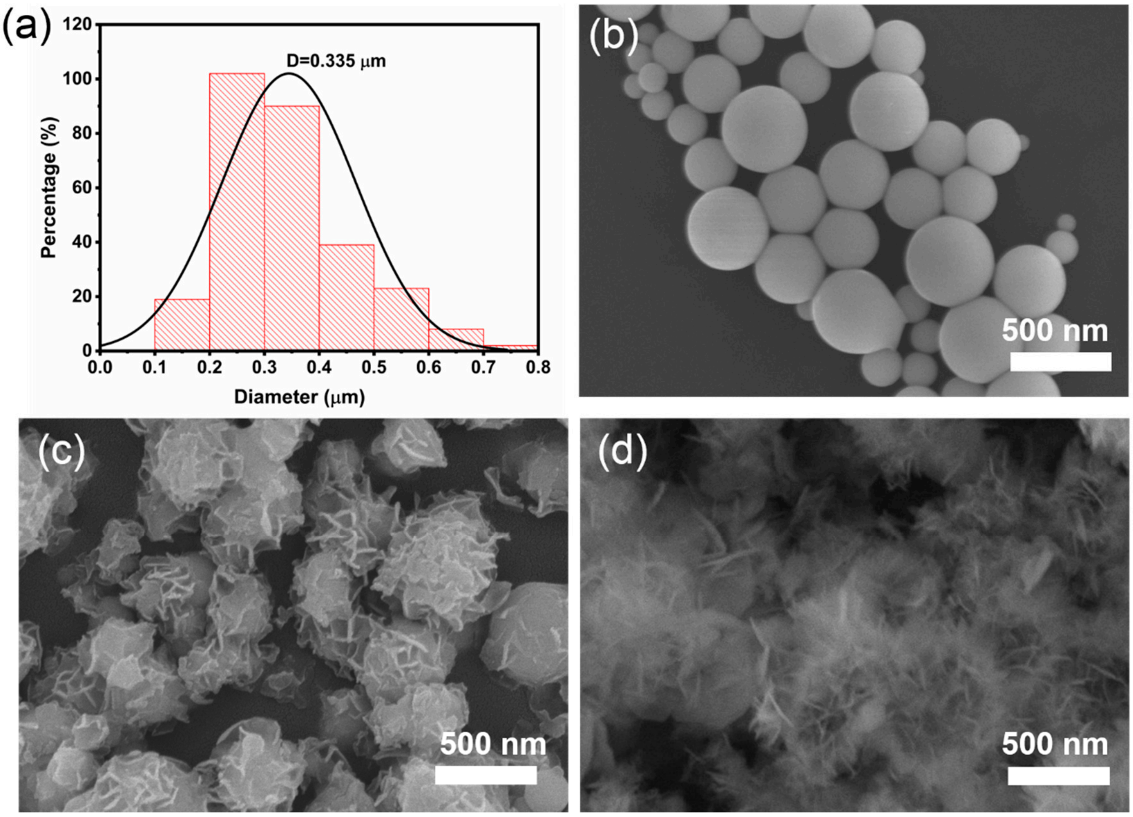

Figure S1. (a) The particle size distributions obtained from Figure 1; the larger magnification SEM images of (b) GaZn nanoparticles; (c) CoOOH/Ga; (d) $Co(OH)_2$.

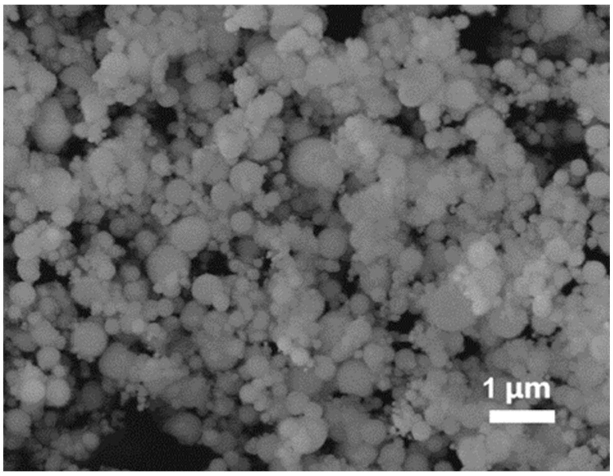

Figure S2. The SEM image of pure Ga nanoparticles reacting with Co solution.

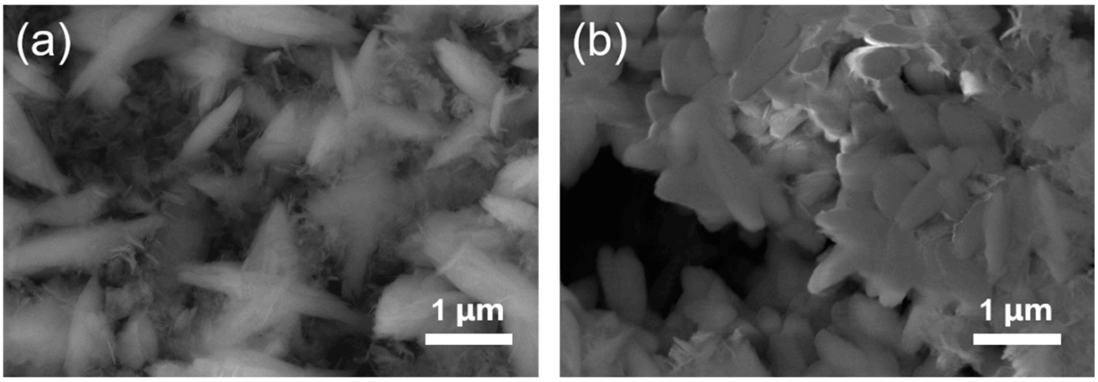

Figure S3. The SEM images of different quality CoOOH/Ga nanoparticles placed in ammonia solution with pH ~ 11.3 (a) $Co(OH)_2$/Ga-40; (b) $Co(OH)_2$/Ga-60.

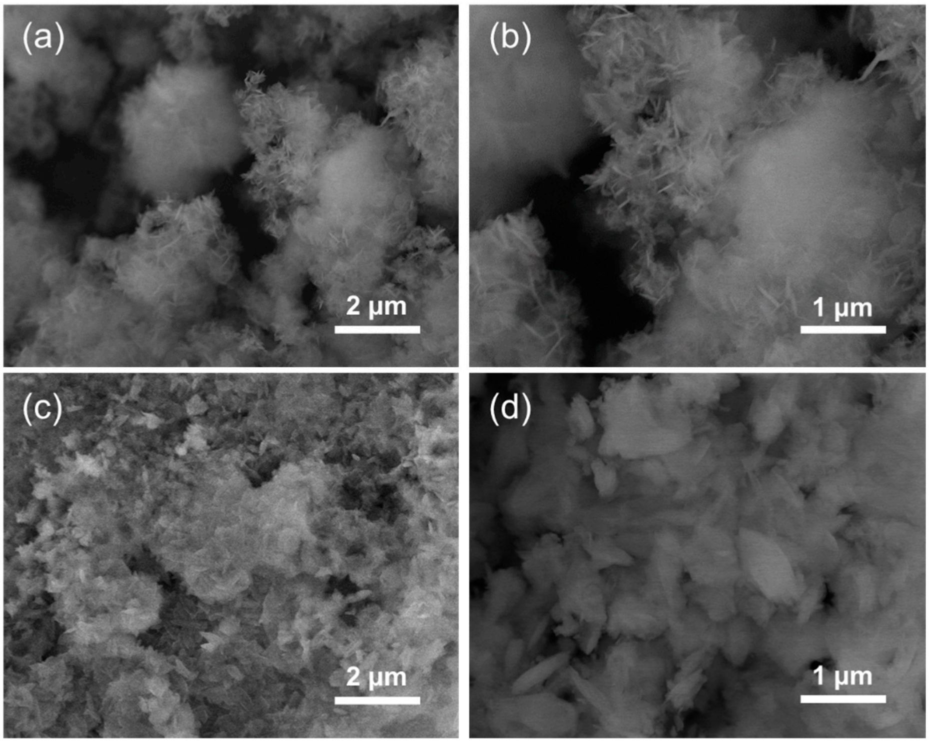

Figure S4. The SEM images of (a, b) the sample of CoOOH/Ga template was cleaned by ammonia solution with pH~12.3; (c, d) the samples of CoOOH/Ga were cleaned by NaOH solution with pH~11.3.

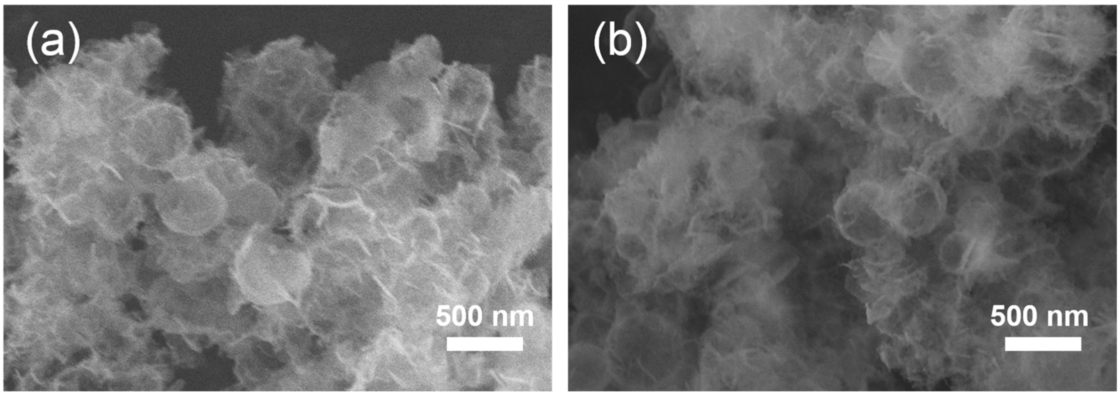

Figure S5. the larger magnification SEM images of (a) $CoSe_2$ and (b) CoS_2.

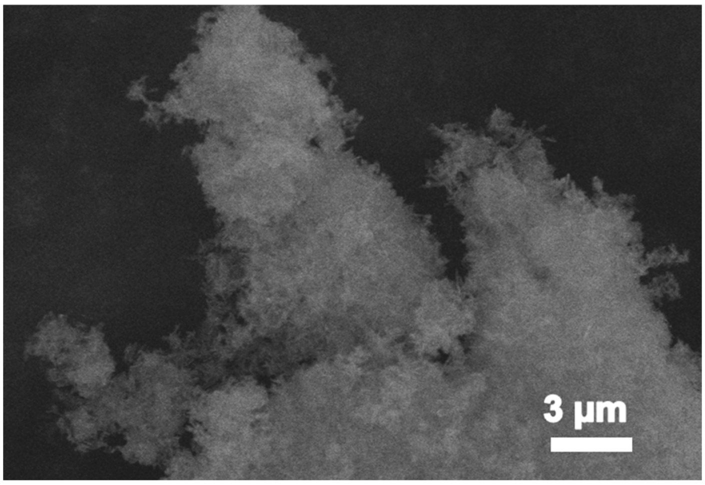

Figure S6. The SEM image of $CoSe_2$-450.

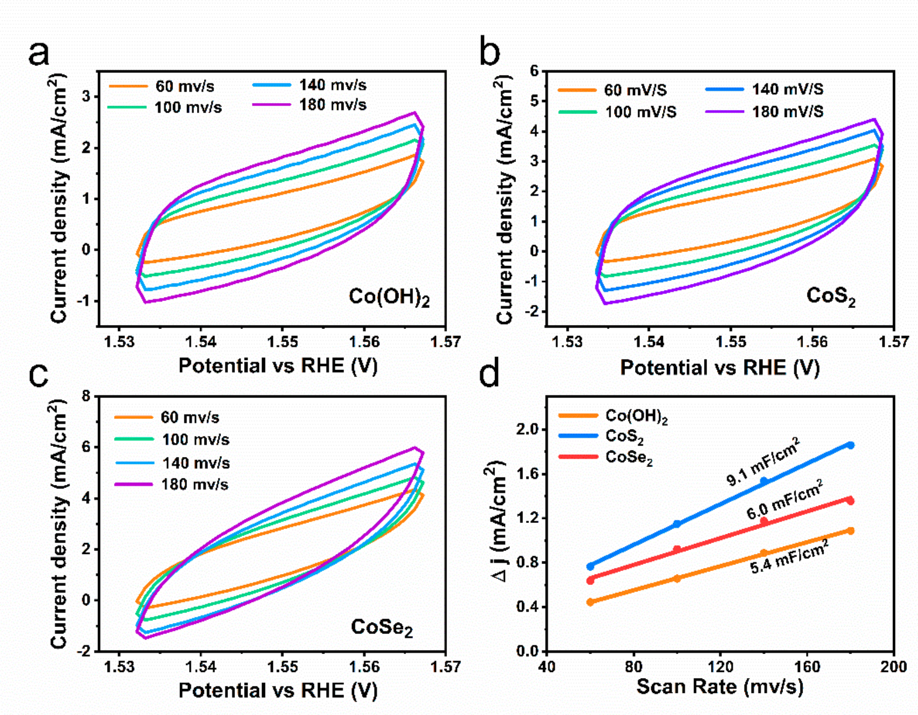

Figure S7. Electrochemical capacitance measurements with different scan rates from 60 to 180 mV/s in 1 M KOH solution of (a) $Co(OH)_2$; (b) CoS_2; (c) $CoSe_2$; (d) The double layer capacitance (C_{dl}) results for all samples.

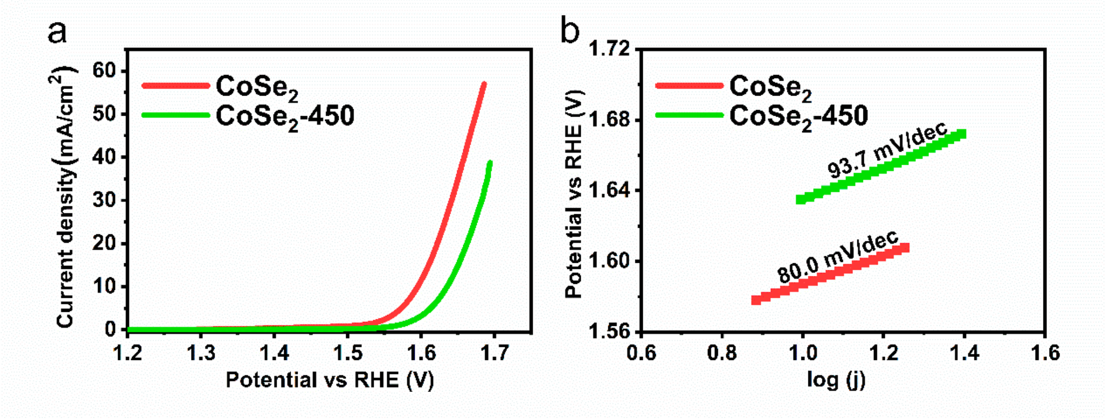

Figure S8. (a) The OER polarization curves and (b) Tafel plots of $CoSe_2$ and $CoSe_2$-450.

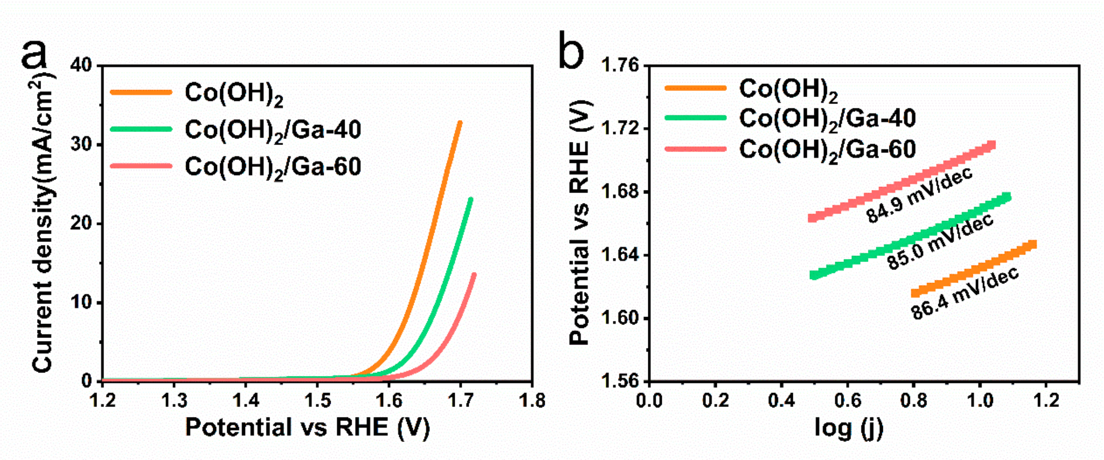

Figure S9. (a) The OER polarization curves and (b) Tafel plots of $Co(OH)_2$, $Co(OH)_2$/Ga-40 and $Co(OH)_2$/Ga-60.
